# Strategies to Reduce Advanced Imaging in Antenatal Pulmonary Embolism Diagnostics

**DOI:** 10.1001/jamanetworkopen.2025.41255

**Published:** 2025-11-04

**Authors:** David R. Vinson, Madeline J. Somers, Lara Zekar, Edward Qiao, Cydney E. Middleton, Sara T. Woldemariam, Nachiketa Gupta, Luke S. Poth, Mary E. Reed, Jeffrey D. Sperling, Nareg H. Roubinian

**Affiliations:** 1 The Permanente Medical Group, Pleasanton, California; 2 Kaiser Permanente Division of Research, Pleasanton, California; 3 Department of Emergency Medicine, Kaiser Permanente Roseville Medical Center, Roseville, California; 4 UC Davis Health, University of California, Davis, Sacramento; 5 California Northstate University School of Medicine, Elk Grove; 6 Department of Obstetrics and Gynecology, Kaiser Permanente Oakland Medical Center, Oakland, California; 7 Department of Emergency Medicine, Kaiser Permanente Redwood City Medical Center, Redwood City, California; 8 Department of Emergency Medicine, Kaiser Permanente South San Francisco Medical Center, South San Francisco, California; 9 Department of Obstetrics and Gynecology, Division of Maternal-Fetal Medicine, Kaiser Permanente Modesto Medical Center, Modesto, California; 10 Department of Pulmonary and Critical Care Medicine, Kaiser Permanente Oakland Medical Center, Oakland, California

## Abstract

**Importance:**

Advanced imaging to diagnose pulmonary embolism (PE) during pregnancy increases costs, ionizing radiation exposure, and the risk of overdiagnosis. D-dimer testing can exclude PE and compression ultrasonography can diagnose deep vein thrombosis (DVT), both reducing advanced imaging needs. How these 2 strategies are used in community PE diagnostics is unknown.

**Objective:**

To analyze the use and efficiencies of D-dimer and ultrasonography for the diagnosis of antenatal PE.

**Design, Setting, and Participants:**

This retrospective cohort study was performed in 21 US community hospitals from October 1, 2021, to March 31, 2023. Participants included pregnant patients who underwent D-dimer testing, compression ultrasonography, or advanced imaging for suspected PE.

**Exposures:**

D-dimer testing or ultrasonography.

**Main Outcomes and Measures:**

The main outcome was avoidance of advanced imaging, measured by the number needed to test (NNT) to avoid 1 advanced imaging study using low to intermediate D-dimer values or a positive ultrasonography finding. Variables associated with D-dimer and ultrasonography were evaluated using quasi-Poisson regression reported as adjusted relative risks (ARRs).

**Results:**

A total of 720 outpatient encounters in 699 patients were included in the analysis (median age, 30.2 [IQR, 24.3-36.1] years). D-dimer testing was obtained in 524 patients (72.8%). Advanced imaging was not pursued in 149 of 155 patients (96.1%) with low (<0.5 mg/L) and 82 of 179 (45.8%) with intermediate (0.5 to <1.0 mg/L) D-dimer values, yielding an NNT to avoid 1 advanced imaging study of 2.3 (95% CI, 2.0-2.6). No 90-day venous thromboembolism or death occurred following rule-outs. D-dimer testing was less commonly used in third (vs first) trimester (ARR, 0.85; 95% CI, 0.75-0.97); with unilateral signs or symptoms of DVT (vs none) (ARR, 0.67; 95% CI, 0.54-0.82), and during labor and delivery unit (vs emergency department) evaluation (ARR, 0.27; 95% CI, 0.20-0.35). Compression ultrasonography was performed in 229 patients (31.8%), with DVT diagnosed in 3 (1.3%). Advanced imaging was not pursued in 2 of these patients, yielding an NNT to avoid 1 advanced imaging study of 115 (95% CI, 32-417). Ultrasonography use and results varied by DVT symptoms. Among 55 patients with DVT symptoms, 46 (83.6%) underwent ultrasonography, with positive findings in 3 (yield of 6.5%). Among 665 patients without symptoms, 183 (27.5%) underwent ultrasonography, with negative findings in all (yield of 0).

**Conclusions and Relevance:**

In this cohort study, use of D-dimer testing was efficient and safe with opportunities for expansion across settings and patients. These findings suggest that ultrasonography efficiency was low but could be improved with symptom-driven use in antenatal PE diagnostics.

## Key Points

**Question:**

How are evidence-based strategies to reduce use of advanced imaging for pregnant patients undergoing pulmonary embolism (PE) diagnostics applied in community practice?

**Findings:**

This cohort study of 720 patient encounters across 21 hospitals in an integrated health system found substantial underuse of D-dimer to safely rule out PE in labor and delivery units compared with emergency departments (19 of 84 [23%] vs 504 of 620 [81%]) and in third-trimester compared with first-trimester patients (159 of 268 [59%] vs 135 of 159 [85%]). Compression ultrasonography was overused in patients without leg symptoms with low yield (0 of 183).

**Meaning:**

These findings suggest that opportunities exist to improve efficient and effective use of guideline-recommended imaging-reducing strategies in antenatal PE diagnostics.

## Teaser

This cohort study describes the prevalence and yield of D-dimer testing to rule out pulmonary embolism and compression ultrasonography to rule in venous thromboembolism without use of advanced imaging among pregnant outpatients.

## Introduction

Pulmonary vascular imaging (henceforth termed *advanced imaging*) is central to the diagnosis of antenatal pulmonary embolism (PE) and commonly includes computed tomography pulmonary angiography (CTPA) and lung scintigraphy, using perfusion alone or ventilation-perfusion imaging.^1,2,3,4,5^ Overimaging in PE diagnostics, however, is common.^6,7,8^ Obtaining advanced imaging in patients with very low probability of PE increases costs, length of stay, risk of overdiagnosis, and ionizing radiation exposure to the patient and fetus.^6,8,9,10,11,12,13^

Two complementary strategies can safely direct advanced imaging to pregnant patients with suspected PE who are most likely to benefit.^6^ One strategy links D-dimer values with risk stratification scores to safely rule out PE without advanced imaging. This approach is founded on 2 seminal studies^14,15,16,17^ published in 2018 and 2019 that reduced advanced imaging by 12% to 39% (eTable 1 in Supplement 1). The second strategy uses compression ultrasonography to evaluate for deep vein thrombosis (DVT), which, when findings are positive, rules in venous thromboembolism (VTE). This indicates anticoagulation and often renders pulmonary vascular imaging unnecessary.^3,4,5^ Because the yield of compression ultrasonography varies significantly by DVT symptoms, nearly all society guidelines reserve extremity imaging for the minority of patients with suspected PE who have suggestive DVT signs and symptoms.^1,3,18,19,20^ However, the overall reduction in advanced imaging for PE from diagnosing DVT is much smaller than that from D-dimer–based algorithms.^20^

The uptake of these 2 strategies to reduce advanced imaging in community practice is unknown, as are their effectiveness and safety. In this cohort study, we described the prevalence and yield of D-dimer testing to rule out PE and compression ultrasonography to rule in VTE without advanced imaging among pregnant outpatients undergoing PE diagnostics across 21 US community medical centers in an integrated health system in 2021 to 2023, several years after publication of the D-dimer–based seminal studies.^14,15,16,17^ We evaluated safety of the D-dimer–based rule-out strategy via the 90-day diagnostic failure rate^21^ and all-cause mortality.

## Methods

### Study Design and Setting

The Antenatal PE Diagnostics (APED) study is a retrospective cohort study undertaken across 21 community medical centers and associated clinics of Kaiser Permanente (KP) Northern California.^13,22,23^ The health system cares for more than 4.5 million members. KP health plan members have similar demographic and socioeconomic characteristics to the local and state populations.^24,25^ KP Northern California is supported by an integrated electronic health record (EHR) that includes all inpatient and outpatient data.^26^ There were no clinical decision support systems or algorithms in place for antenatal PE diagnostics throughout the study period. Diagnostic testing was at the clinicians’ discretion. Compression ultrasonography from radiology was routinely available for patients in the emergency department (ED) and labor and delivery unit (LDU) daily from 7 am to 9 pm and lung scintigraphy daily from 8 am to 5 pm. Off-hours availability was limited and varied by facility. All study centers used a rapid, automated, quantitative immuno-turbidimetric D-dimer assay (STA-Liatest D–Di; Stago).^27^

The Institutional Review Board of KP Northern California approved the study and waived informed consent for the collection of retrospective EHR data. We followed the Strengthening the Reporting of Observational Studies in Epidemiology (STROBE) reporting guideline.

### Population

The larger APED study population consisted of 2144 pregnant adults (aged ≥18 years) who underwent at least 1 of 4 VTE diagnostic tests (D-dimer, compression ultrasonography, CTPA, or lung scintigraphy) for suspected PE in the ED, LDU, or clinic setting from October 1, 2021, through March 31, 2023.^13,22,23^ We identified pregnant persons using the KP Northern California Division of Research Perinatal Research Unit’s Perinatal Obstetric Database. We used procedural codes to identify testing and imaging recipients, which we confirmed during manual EHR review, as with prior PE studies.^28,29^ We included cases with compression ultrasonography only if the procedure was completed independently or prior to ordering advanced imaging, because we sought to measure the association of ultrasonography results with advanced imaging ordering. We manually reviewed EHRs of pregnant patients to confirm that clinicians suspected PE and that patients had at least 1 of the following PE-related symptoms: new or worsening dyspnea, chest or thoracic pain, hemoptysis, syncope, presyncope,^30^ or palpitations. We excluded patients who underwent testing for reasons other than PE diagnosis (eg, for DVT diagnostics in patients without suspected PE). We excluded those not known to be pregnant at the time of diagnostics, whose diagnostic evaluation began as an inpatient, who had recent or impending pregnancy loss, who had known VTE treated with anticoagulation, or who left before completing the agreed diagnostic evaluation. We also excluded patients with COVID-19, as it could alter the indications for and selection of D-dimer and advanced imaging testing.^31,32,33^

### Outcomes

Our primary outcome was nonpursuit of advanced imaging following a low to intermediate D-dimer value (<1.0 mg/L [to convert to nmol/L, multiply by 5.476]) (eTable 1 in Supplement 1) or compression ultrasonography of the proximal lower extremity (or upper extremity if indicated) positive for DVT.^14,15^ Advanced imaging that was recommended but declined by a patient was categorized as pursued.^9,13^ We measured efficiency of these 2 strategies by the number needed to test (NNT) to avoid pursuit of 1 advanced imaging study.^34^

Safety outcomes of D-dimer testing without advanced imaging were (1) the diagnostic failure rate, defined as the 90-day incidence of adjudicated VTE,^21,35^ and (2) the 90-day incidence of adjudicated all-cause mortality. We measured mortality using the health system mortality database that links to the Social Security death master file and the California State Department of Vital Statistics. We searched the KP Northern California claims database to identify outcomes outside the health care system.

### Use of D-Dimer Tests

We reported advanced imaging by D-dimer strata, using thresholds from the Artemis study^15^: less than 0.5 mg/L indicated low, negative, or normal; 0.5 to less than 1.0 mg/L, intermediate; and 1.0 mg/L or greater, high (eTable 1 in Supplement 1). To estimate pretest probability, we used the pregnancy-adapted Geneva score, which is amenable to retrospective calculation as it is comprised exclusively of routinely documented objective variables, unlike the YEARS criteria (eTables 1 and 2 in Supplement 1).^36,37^

### Data Collection

Study data were compiled using automated data sources extracted from the EHR and data abstracted during manual EHR review, as reported in prior APED publications (eMethods 1 in Supplement 1).^13,22,23^ We defined low socioeconomic status using residential census block group with 25% or more of adult residents having less than a high school education or 20% or more of households having an annual income below the federal poverty level.^38^ We calculated turnaround times using structured time stamps in the clinical databases to evaluate the time costs of respective diagnostic testing. We estimated costs of the 2 diagnostic tests using the 2022 fee schedule of the US Centers for Medicare & Medicaid Services, based on *Current Procedural Terminology* codes 93970 and 93971 for bilateral and unilateral compression ultrasonography, respectively, and 85379 for quantitative D-dimer testing.^39,40^

Race and ethnicity were self-reported and were included to reflect the diversity of the population of northern California. Categories included Asian, Black or African American, Hispanic or Latino, non-Hispanic White, and other (including American Indian or Alaska Native, Native Hawaiian or Other Pacific Islander, and declined to state).

### Statistical Analysis

We presented continuous variables as medians with IQRs and categorical data as frequencies and proportions. We conducted analyses at the encounter rather than the patient level given repeated measures. We reported the number and prevalence of missing variables. We used quasi-Poisson regression to calculate adjusted relative risks (ARRs) of each imaging-reducing strategy (D-dimer testing and compression ultrasonography) with 95% CIs. The selection of variables for modeling is explained in eMethods 2 in Supplement 1. In a sensitivity analysis, we undertook the regressions without repeat visits for diagnostic testing for suspected PE. *P* values were calculated using the Pearson χ^2^ test, Wilcoxon rank sum test, or Fisher exact test as indicated. A 2-sided *P* < .05 was considered statistically significant. Interrater reliability has been previously reported in 2 related APED studies.^13,22^ Data management was conducted in SAS, version 9.4 (SAS Institute Inc), and all analyses were conducted in R, version 4.3.1 (R Program for Statistical Computing).

## Results

Among 2144 pregnant adults who underwent at least 1 of 4 VTE diagnostic tests during the 18-month study period, we excluded 1280 from complete EHR review, primarily because testing was not undertaken for PE diagnostics (Figure). After complete EHR review, we excluded an additional 144 patients with COVID-19. The remaining 720 outpatient encounters among 699 individual patients constitute the study cohort and were categorized by advanced imaging–reducing strategies (Figure). The median age of the cohort was 30.2 (IQR, 24.3-36.1) years (Table 1). Overall, 620 encounters (86.1%) were evaluated in the ED, 84 (11.7%) in the LDU, and 16 (2.2%) in outpatient clinics (14 obstetric and 2 nonobstetric). Overall, 315 encounters (43.8%) included advanced imaging: initial CTPA in 288 (91.4%) and initial lung scintigraphy in 27 (8.6%). Five of these (1.6%) resulted in a diagnosis of acute PE, all by CTPA. In terms of race and ethnicity, 119 patients (16.5%) were Asian, 95 (13.2%) were Black or African American, 257 (35.7%) were Hispanic or Latino, 212 (29.4%) were non-Hispanic White, and 37 (5.1%) were of other race or ethnicity.

**Figure.**
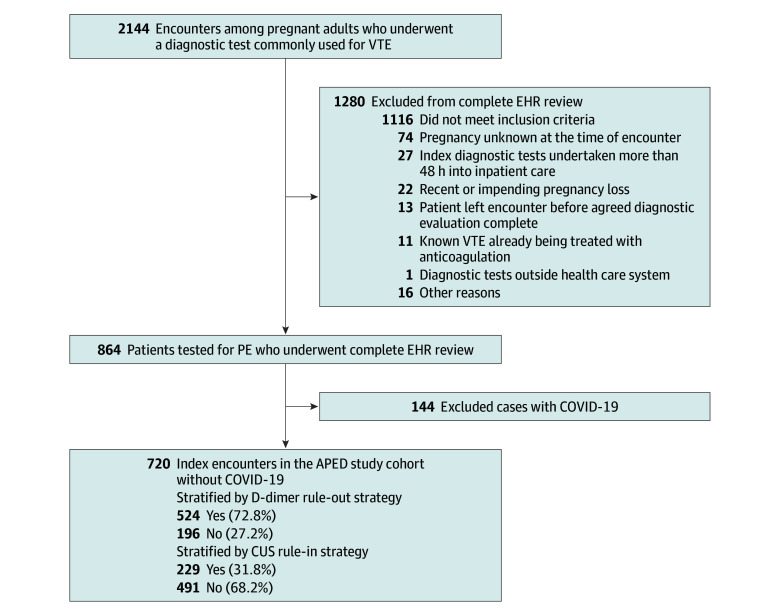
Cohort Assembly and Strategies to Reduce Advanced Imaging APED indicates Antenatal Pulmonary Embolism Diagnostics; CUS, compression ultrasonography; EHR, electronic health record; PE, pulmonary embolism; VTE, venous thromboembolism.

**Table 1.** Characteristics of Pregnant Patients Undergoing Diagnostic Testing for Acute PE, Stratified by Strategies to Reduce Advanced Imaging

Characteristic	Overall, No. (%) (N = 720)^b^	Use of D-dimer testing, No. (%)^a^		*P* value	Use of compression ultrasonography, No. (%)^a^		*P* value
		Yes (n = 524 [72.8])	No (n = 196 [27.2])		Yes (n = 229 [31.8])	No (n = 491 [68.2])	
Age, median (IQR), y	30.0 (26.0-35.0)	30.0 (25.0-34.0)	32.0 (27.0-35.0)	.02	31.0 (26.0-34.0)	30.0 (26.0-35.0)	.74
Age category, y							
<40	684 (95.0)	499 (73.0)	185 (27.0)	.64	219 (32.0)	465 (68.0)	.59
≥40	36 (5.0)	25 (69.4)	11 (30.6)		10 (27.8)	26 (72.2)	
Race and ethnicity							
Asian	119 (16.5)	76 (63.9)	43 (36.1)	.02	48 (40.3)	71 (59.7)	.05
Black or African American	95 (13.2)	75 (78.9)	20 (21.1)		23 (24.2)	72 (75.8)	
Hispanic or Latino	257 (35.7)	192 (74.7)	65 (25.3)		87 (33.9)	170 (66.1)	
Non-Hispanic White	212 (29.4)	156 (73.6)	56 (26.4)		58 (27.4)	154 (72.6)	
Other^c^	37 (5.1)	25 (67.6)	12 (32.4)		13 (35.1)	24 (64.9)	
Insurance category							
Commercial	555 (77.1)	399 (71.9)	156 (28.1)	.33	173 (31.2)	382 (68.8)	.50
Medicaid	165 (22.9)	125 (75.8)	40 (24.2)		56 (33.9)	109 (66.1)	
Socioeconomic status							
Not low	499 (69.3)	352 (70.5)	147 (29.5)	.06	158 (31.7)	341 (68.3)	.57
Low	177 (24.6)	138 (78.0)	39 (22.0)		52 (29.4)	125 (70.6)	
Unknown	44 (6.1)	34 (77.3)	10 (22.7)		19 (43.2)	25 (56.8)	
Prepregnancy BMI							
<30	341 (47.4)	248 (72.7)	93 (27.3)	.39	112 (32.8)	229 (67.2)	.94
30-39	208 (28.9)	143 (68.8)	65 (31.3)		69 (33.2)	139 (66.8)	
≥40	45 (6.3)	29 (64.4)	16 (35.6)		16 (35.6)	29 (64.4)	
Unknown	126 (17.5)	104 (82.5)	22 (17.5)		32 (25.4)	94 (74.6)	
Gravidity							
1	178 (24.7)	127 (71.3)	51 (28.7)	.63	59 (33.1)	119 (66.9)	.67
≥2	541 (75.1)	396 (73.2)	145 (26.8)		170 (31.4)	371 (68.6)	
Unknown	1 (0.1)	1 (100)	0		0	1 (100)	
Parity							
0	274 (38.1)	199 (72.6)	75 (27.4)	.94	83 (30.3)	191 (69.7)	.49
≥1	446 (61.9)	325 (72.9)	121 (27.1)		146 (32.7)	300 (67.3)	
History of VTE	34 (4.7)	21 (61.8)	13 (38.2)	.14	14 (41.2)	20 (58.8)	.23
Current gestational diabetes	23 (3.2)	15 (65.2)	8 (34.8)	.41	8 (34.8)	15 (65.2)	.76
Gestational age in trimester (wk)							
First (<14)	159 (22.1)	135 (84.9)	24 (15.1)	<.001	37 (23.3)	122 (76.7)	.01
Second (14-27)	293 (40.7)	230 (78.5)	63 (21.5)		92 (31.4)	201 (68.6)	
Third (≥28)	268 (37.2)	159 (59.3)	109 (40.7)		100 (37.3)	168 (62.7)	
VTE symptoms (not exclusive)							
Shortness of breath	532 (73.9)	375 (70.5)	157 (29.5)	.02	181 (34.0)	351 (66.0)	.03
Thoracic pain	474 (65.8)	359 (75.7)	115 (24.3)	.01	140 (29.5)	334 (70.5)	.07
Palpitations	156 (21.7)	120 (76.9)	36 (23.1)	.19	44 (28.2)	112 (71.8)	.28
Unilateral leg symptoms	55 (7.6)	27 (49.1)	28 (50.9)	<.001	46 (83.6)	9 (16.4)	<.001
Syncope or presyncope	54 (7.5)	43 (79.6)	11 (20.4)	.24	17 (31.5)	37 (68.5)	.96
Hemoptysis	11 (1.5)	8 (72.7)	3 (27.3)	>.99	1 (9.1)	10 (90.9)	.19
Duration of VTE symptoms, h							
<48	444 (61.7)	341 (76.8)	103 (23.2)	.003	138 (31.1)	306 (68.9)	.51
≥48	269 (37.4)	179 (66.5)	90 (33.5)		90 (33.5)	179 (66.5)	
Unknown	7 (1.0)	4 (57.1)	3 (42.9)		1 (14.3)	6 (85.7)	
Heart rate, beats/min							
<110	473 (65.7)	346 (73.2)	127 (26.8)	.76	153 (32.3)	320 (67.7)	.67
≥110	247 (34.3)	178 (72.1)	69 (27.9)		76 (30.8)	171 (69.2)	
Pulse oximetry, %							
95-100	678 (94.2)	495 (73.0)	183 (27.0)	.59	213 (31.4)	465 (68.6)	.48
<95	35 (4.9)	27 (77.1)	8 (22.9)		13 (37.1)	22 (62.9)	
Unknown	7 (1.0)	2 (28.6)	5 (71.4)		3 (42.9)	4 (57.1)	
Site of evaluation							
ED	620 (86.1)	504 (81.3)	116 (18.7)	<.001	194 (31.3)	426 (68.7)	.10
LDU	84 (11.7)	19 (22.6)	65 (77.4)		26 (31.0)	58 (69.0)	
Outpatient clinic	16 (2.2)	1 (6.3)	15 (93.8)		9 (56.3)	7 (43.8)	
Pretest probability of PE^d^							
Low	414 (57.5)	315 (76.1)	99 (23.9)	.07	117 (28.3)	297 (71.7)	.003
Intermediate	280 (38.9)	191 (68.2)	89 (31.8)		97 (34.6)	183 (65.4)	
High	26 (3.6)	18 (69.2)	8 (30.8)		15 (57.7)	11 (42.3)	
Nonconsent to recommended advanced imaging	91 (12.6)	74 (81.3)	17 (18.7)	.05	46 (50.5)	45 (49.5)	<.001

Abbreviations: BMI, body mass index (calculated as weight in kilograms divided by height in meters squared); ED, emergency department; LDU, labor and delivery unit; PE, pulmonary embolism; VTE, venous thromboembolism.

^a^ Percentages are calculated based on row totals.

^b^ Percentages are calculated based on column totals.

^c^ Includes American Indian or Alaska Native (n = 4), Native Hawaiian or Other Pacific Islander (n = 12), and declined to state (n = 21).

^d^ Calculated using the pregnancy adapted Geneva score; 0 to 1 indicates low; 2 to 6, intermediate; and 7 or greater, high.

### D-Dimer Testing and Advanced Imaging

D-dimer use was observed in 524 patient encounters (72.8%). Of these, values were low in 155 (29.6%), intermediate in 179 (34.2%), and high in 190 (36.3%). D-dimer values increased with increasing gestational age (Table 2). Forgoing pursuit of advanced imaging correlated inversely with D-dimer levels (149 of 155 [96.1%] with low [<0.5 mg/L] and 82 of 179 [45.8%] with intermediate [0.5 to <1.0 mg/L] values). The NNT to avoid 1 advanced imaging study was 2.3 (95% CI, 2.0-2.6), calculated by dividing the total number of D-dimer tests performed (n = 524) by the number of cases with low to intermediate D-dimer values in which advanced imaging was not pursued (n = 231). We did not include in the denominator nonpursuit of advanced imaging in patients with high D-dimer values, as advanced imaging is recommended for this population (eTable 1 in Supplement 1).^14,15,16,17^

**Table 2.** D-Dimer Orders and Results in Pregnant Patients With Suspected Pulmonary Embolism, Stratified by Trimester

D-dimer data	Trimester, No. (%)^a^		
	First (n = 159)	Second (n = 293)	Third (n = 268)
D-dimer measurement obtained			
No	24 (15.1)	63 (21.5)	109 (40.7)
Yes	135 (84.9)	230 (78.5)	159 (59.3)
D-dimer value, mg/L			
<0.5	86 (63.7)	66 (28.7)	3 (1.9)
≥0.5 and <1.0	31 (23.0)	103 (44.8)	45 (28.3)
≥1.0	18 (13.3)	61 (26.5)	111 (69.8)

SI conversion factor: To convert D-dimer to µmol/L, multiply by 5.476.

^a^ Calculated based on column totals.

D-dimer–based imaging patterns were similar between the ED setting and the LDU setting (eTable 4 in Supplement 1). We found pursuit of advanced imaging increased with increasing pretest probability scores for those with intermediate D-dimer values (eTable 5 in Supplement 1). No 90-day VTE or mortality occurred following use of low to intermediate D-dimer values to rule out PE without advanced imaging.

### D-Dimer Testing Patterns

D-dimer use varied significantly by setting, including 504 of 620 patients (81.3%) in the ED, 19 of 84 (22.6%) in the LDU, and 1 of 16 (6.3%) in the clinic (*P* < .001). D-dimer use decreased as trimesters increased (third trimester, 159 of 268 [59.3%]; first trimester, 135 of 159 [84.9%]; *P* < .001) (Table 1). The following were associated with lower rates of D-dimer testing: third vs first trimester (ARR, 0.85; 95% CI, 0.75-0.97), unilateral signs or symptoms of DVT vs none (ARR, 0.67; 95% CI, 0.54-0.82), and LDU (ARR, 0.27; 95% CI, 0.20-0.35) or outpatient clinic (ARR, 0.12; 95% CI, 0.03-0.29) vs ED setting (Table 3).

**Table 3.** Adjusted Relative Risks of D-Dimer Testing as a Strategy to Reduce Advanced Imaging During Antenatal PE Diagnostics^a^

Characteristic	ARR (95% CI)	*P* value
Age ≥40 (vs <40) y	1.00 (0.80-1.23)	>.99
Race and ethnicity		
Asian	0.90 (0.77-1.04)	.30
Black or African American	1.09 (0.93-1.27)	
Hispanic or Latino	1.01 (0.89-1.13)	
Non-Hispanic White	1 [Reference]	
Other^b^	0.95 (0.75-1.19)	
Low socioeconomic status (vs not low)	1.03 (0.92-1.14)	.65
Gestational age, trimester (wk)		
First (<14)	1 [Reference]	.02
Second (14-27)	0.99 (0.88-1.11)	
Third (≥28)	0.85 (0.75-0.97)	
History of VTE (vs none)	0.82 (0.64-1.03)	.10
Syncope or presyncope (vs none)	0.98 (0.82-1.15)	.79
Unilateral signs or symptoms (vs none) of DVT^c^	0.67 (0.54-0.82)	<.001
Maximum heart rate ≥110 (vs <110) beats/min	1.05 (0.95-1.16)	.36
Lowest pulse oximetry <95% (vs ≥95%)	1.11 (0.88-1.36)	.37
Site of evaluation		
ED	1 [Reference]	<.001
LDU	0.27 (0.20-0.35)	
Outpatient clinic	0.12 (0.03-0.29)	
Nonconsent to recommended advanced imaging	1.01 (0.88-1.16)	.86

Abbreviations: ARR, adjusted relative risk; DVT, deep vein thrombosis; ED, emergency department; LDU, labor and delivery unit; PE, pulmonary embolism; VTE, venous thromboembolism.

^a^ Only socioeconomic status had missing values: (44 of 720 [6.1%]). This was unlikely to have greatly skewed the results.

^b^ Includes American Indian or Alaska Native, Native Hawaiian or Other Pacific Islander, and decline to state.

^c^ Signs and symptoms are distinguished in the pregnancy-adapted Geneva score and have been combined here.

Among cases undergoing D-dimer testing, clinicians documented use of a risk tool in 107 of 524 (20.4%), including YEARS in 87 (16.6%), Wells in 18 (3.4%), and revised Geneva score in 2 (0.4%). Documentation of risk scores was more prevalent in ED (n = 105) vs non-ED (n = 2) settings. Turnaround time from order to result of D-dimer testing was slightly shorter in the ED setting than in the LDU setting (eTable 3 in Supplement 1).

Clinicians did not pursue advanced imaging in 13 of 155 patients (8.4%) with high D-dimer values (≥1.0 mg/L) and absent or negative ultrasonography findings. In 12 of these 13 cases, the high D-dimer values were less than 2.0 mg/L. In 8 of these cases, treating clinicians documented their perception that the elevation was normal for third-trimester pregnancies. No 90-day VTE or mortality was documented among patients with high D-dimer values who did not undergo advanced imaging.

### Compression Ultrasonography and Advanced Imaging

Overall, 229 patients (31.8%) underwent compression ultrasonography prior to or independently of advanced imaging (182 bilateral and 37 unilateral). Twenty-nine additional ultrasonograms were not completed prior to ordering advanced imaging and therefore were not included in the analysis (details are given in eTable 6 in Supplement 1). Forty-six of 55 patients (83.6%) with and 183 of 665 (27.5%) without unilateral DVT symptoms underwent ultrasonography. Among ultrasonography recipients, DVT diagnosis was low (3 of 229 [1.3%]) and varied between 3 of 46 (6.5%) with vs 0 of 183 without symptoms (*P* = .09). In 2 of 3 cases of pregnant patients with suspected PE who had compression ultrasonography positive for DVT, the clinician did not pursue advanced imaging. The NNT for ultrasonography to avoid 1 case of advanced imaging was 115 (95% CI, 32-417 [229/2]), significantly higher than the NNT with D-dimer use (2.3). The NNT would have been much lower (23; 95% CI, 7-170 [46/2]) if ultrasonography was not used in 183 patients without DVT symptoms.

Overall, 106 of 229 ultrasonography recipients (46.3%) were recommended to receive advanced imaging: 1 patient with positive ultrasonography findings and 105 patients with negative ultrasonography findings. Nonpursuit of pulmonary vascular imaging was common among patients with negative compression ultrasonography (121 of 226 [53.5%]). Nonpursuit of advanced imaging was more common among patients with than without DVT symptoms (31 of 43 [72.1%] vs 90 of 183 [49.2%]). D-dimer testing was performed in 60 of 121 patients (49.6%) whose clinicians failed to pursue advanced imaging after negative ultrasonography findings. Among this subset of 60 patients, D-dimer values were elevated (>0.5 µg/mL) in 35 (58.3%), 7 of whom had values of 1.0 µg/mL or greater.

### Compression Ultrasonography Patterns of Use

The prevalence of compression ultrasonography was similar across ED and LDU settings (Table 1). After adjusting for demographic and select clinical variables, the following were associated with ultrasonography: third vs first trimester (ARR, 1.48; 95% CI, 1.05-2.09), DVT symptoms vs none (ARR, 3.00; 95% CI, 2.24-3.96), and not consenting vs consenting to advanced imaging (ARR, 1.79; 95% CI, 1.33-2.38) (Table 4). Turnaround times of ultrasonography were slightly shorter in the ED than in the LDU; both were much shorter than in outpatient clinics (eTable 3 in Supplement 1).

**Table 4.** Adjusted Relative Risks of Compression Ultrasonography as a Strategy to Reduce Advanced Imaging During Antenatal PE Diagnostics^a^

Characteristic	ARR (95% CI)	*P* value
Age ≥40 (vs <40) y	1.11 (0.60-1.87)	.73
Race and ethnicity		
Asian	1.39 (0.98-1.97)	.27
Black or African American	0.91 (0.58-1.40)	
Hispanic or Latino	1.19 (0.87-1.63)	
Non-Hispanic White	1 [Reference]	
Other^b^	1.09 (0.61-1.84)	
Low socioeconomic status (vs not low)	0.96 (0.72-1.25)	.75
Gestational age, trimester (weeks)		
First (<14)	1 [Reference]	.08
Second (14-27)	1.26 (0.91-1.77)	
Third (≥28)	1.48 (1.05-2.09)	
History of VTE (vs none)	1.46 (0.87-2.28)	.14
Syncope or presyncope (vs none)	1.28 (0.80-1.93)	.29
Unilateral signs or symptoms (vs none) of DVT^c^	3.00 (2.24-3.96)	<.001
Maximum heart rate ≥110 (vs <110) beats/min	1.01 (0.78-1.29)	.95
Lowest pulse oximetry <95% (vs ≥95%)	1.09 (0.61-1.79)	.76
Site of evaluation		
ED	1 [Reference]	.42
LDU	0.91 (0.61-1.33)	
Outpatient clinic	1.58 (0.73-2.97)	
Nonconsent to recommended advanced imaging	1.79 (1.33-2.38)	<.001

Abbreviations: ARR, adjusted relative risk; DVT, deep vein thrombosis; ED, emergency department; LDU, labor and delivery unit; PE, pulmonary embolism; VTE, venous thromboembolism.

^a^ Only socioeconomic status had missing values: (44 of 720 [6.1%]). This was unlikely to have greatly skewed the results.

^b^ Includes American Indian or Alaska Native, Native Hawaiian or Other Pacific Islander, and decline to state.

^c^ Signs and symptoms are distinguished in the pregnancy-adapted Geneva score and have been combined here.

### Repeat Visits, Costs, and Comparison of Strategies

There was a nominal number of repeat visits for diagnostic testing for suspected PE (21 [2.9%]). In sensitivity analyses, we ran the D-dimer and ultrasonography regression models after dropping subsequent visits for these 21 individuals (ie, 699 individual patients). No meaningful changes in significance or in associations were noted. Calculations of US cost estimates are provided in the eResults and eTable 7 in Supplement 1; features of both strategies to reduce advanced imaging are provided in eTable 8 in Supplement 1.

## Discussion

In this retrospective cohort study of pregnant patients in 21 US community medical centers, 2 complementary strategies were commonly used to reduce the need for advanced imaging. D-dimer testing to rule out PE was used in almost three-fourths of patients, with lower use in the third trimester, among patients with DVT signs or symptoms, and in non-ED settings. Compression ultrasonography to rule in VTE was used in nearly one-third of patients with suspected PE, with higher use in patients with DVT symptoms and in those who declined recommended advanced imaging. The 2 strategies varied in efficiency, as measured by the NNT to avoid 1 advanced imaging study (2.3 for D-dimer vs 115 for ultrasonography) and varied significantly in costs.

D-dimer testing should be used in all (or nearly all) gravid patients undergoing diagnostic testing for PE to identify those who may safely forgo advanced imaging.^3,4,41^ In the prospective CT-PE-Pregnancy study of the performance of a D-dimer–based revised Geneva algorithm, D-dimer evaluation was indicated in all patients with low to intermediate pretest probability, which encompassed 354 of 357 patients (99.2%) in the study cohort.^14^ In Artemis, a prospective management study of the performance of a D-dimer–based pregnancy-adapted YEARS algorithm, D-dimer testing was indicated in all patients with suspected PE (eTable 1 in Supplement 1).^15^ Approximately one-quarter of our study patients did not undergo D-dimer testing. Why the presence of DVT symptoms was independently associated with reduced D-dimer testing is unclear.

Had D-dimer results been available for the entire cohort, advanced imaging may not have been indicated in some for whom it was recommended, and it may have been indicated in some for whom it was not recommended. For example, approximately 20% of pregnant patients who declined advanced imaging did not undergo D-dimer testing.^13^ Some of these nonconsenting patients may have had a low D-dimer value, which may well have avoided the disagreement over advanced imaging.

D-dimer testing use patterns differed remarkably by practice setting. D-dimer testing was more than 3-fold higher in the ED setting compared with the LDU setting. Emergency physicians have long been accustomed to using D-dimer testing for PE diagnostics outside pregnancy and the postpartum period.^42,43,44^ This ED familiarity with D-dimer testing use may have facilitated the transition to using D-dimer testing in pregnancy following the 2 seminal prospective management studies published in 2018 and 2019 that demonstrated the safety and utility of D-dimer testing in antenatal PE diagnostics.^14,15,16,17^ The most recent American College of Obstetricians and Gynecologists Practice Bulletin on VTE diagnostics^1^ was published before these aforementioned studies and recommended against D-dimer testing use (eDiscussion in Supplement 1), as did other society guidelines published prior to 2019. Forthcoming guidelines will be able to engage with more recent studies of D-dimer use in antenatal PE diagnostics.

D-dimer testing was also less commonly used in later trimesters (84.9% of first vs 59.3% of third-trimester patients). D-dimer values are known to rise with increasing gestational age. Among 494 patients in the Artemis study, median D-dimer levels were 0.51 mg/L during the first trimester, 0.73 mg/L during the second trimester, and 1.1 mg/L during the third trimester.^15^ Because of rising D-dimer levels, the YEARS algorithm safely averted CTPA in fewer patients with increasing trimesters: 65% during the first, 46% of patients during the second, and 32% of patients during the third trimester. Although the algorithm was less effective in the third trimester, it remained an effective strategy. Antenatal PE diagnostics could be improved by expanding D-dimer use across all trimesters.

The yield of compression ultrasonography in diagnosing DVT in pregnant patients with suspected PE was greater in those with than without DVT symptoms (6.5% vs 0, respectively). A recent metanalysis of 482 pregnant patients with suspected PE who underwent ultrasonography also had disparate ultrasonography results^20^: 7.9% positivity in patients with vs 0.8% in those without DVT symptoms. The American College of Obstetricians and Gynecologists, the American Thoracic Society, and the European Society of Cardiology, among others, recommend a selective symptom-driven ultrasonography approach during antenatal PE diagnostics.^1,3,19,41,45^ No guidelines suggest that negative compression ultrasonography findings alone are sufficient to exclude PE, even in patients with DVT symptoms.

### Strengths and Limitations

Strengths of our study include its racially and ethnically diverse population and multicenter community setting, which increase its generalizability. This US study, however, may not reflect practices of other countries.^7^ Outcome capture was excellent in this closed population. This study was limited by its retrospective nature, which we attempted to mitigate by adhering to established guidelines for EHR review–based studies.^46,47^ Physician abstractors, however, were not blinded to patient variables when identifying study outcomes, which is a possible source of bias. We were unable to include patients with PE-related symptoms who failed to undergo any VTE testing. In this observational study, while we accounted for several patient sociodemographic and clinical factors, we cannot rule out unmeasured confounding and cannot draw causal conclusions. Also, because we lacked documentation about whether PE was the most likely diagnosis, we were unable to assess adherence to the pregnancy-adapted YEARS algorithm in patients with intermediate D-dimer values. Our results may not be generalizable to other geographic locations and practice settings with differences, for example, in prevalence of PE and availability of timely, reliable follow-up.

## Conclusions

This cohort study found a moderate uptake of 2 safe strategies to reduce advanced imaging in antenatal PE diagnostics in a US community setting and identified opportunities to increase utility and efficiency: (1) expand use of D-dimer–based algorithms in obstetric settings and undertested patient categories, (2) focus use of compression ultrasonography on patients with suggestive symptoms and signs of DVT, and (3) pursue advanced imaging in patients with suspected PE as indicated by D-dimer values despite negative compression ultrasonography findings. These strategies could improve adherence to evidence-based practices and reduce unnecessary costs, delays, and risks of advanced imaging in pregnancy.

## Notes

Supplement 1. **eTable 1.** Characteristics of the 2 Seminal Studies of D-Dimer–Based Algorithms for Ruling Out Pulmonary Embolism Without Advanced Imaging **eTable 2.** Revised and Pregnancy-Adapted Geneva Scores **eMethods 1. **Details of Data Collection **eMethods 2. **Selection of Variables for Modeling **eTable 3.** Median Turnaround Times of D-Dimer Testing and Ultrasonography From Order to Completion, Stratified by Diagnostic Setting **eTable 4.** Setting-Specific Pursuit of Advanced Imaging, Stratified by D-Dimer Values **eTable 5.** Pretest Probability for Acute Pulmonary Embolism and Pursuit of Advanced Imaging Among Pregnant Patients With Intermediate D-Dimer Values (≥0.5<1.0 mg/L) **eTable 6.** Characteristics of Pregnant Patients Who Underwent Compression Ultrasonography That Was Not Used as a Strategy to Reduce Advanced Imaging Because Advanced Imaging Was Ordered Before Compression Ultrasonography Was Ordered or Completed **eResults**. US Cost Estimates of Strategies to Reduce Advanced Imaging in Antenatal Pulmonary Embolism Diagnostics **eTable 7.** US Federal Reimbursements in Dollars for D-Dimer Testing and Compression Ultrasonography in 2022 and 2024 **eTable 8.** Features of 2 Strategies to Reduce Advanced Imaging in Antenatal Pulmonary Embolism Diagnostics **eDiscussion.** D-Dimer Use Discouraged by the 2018 American College of Obstetricians and Gynecologists Practice Bulletin

Supplement 2.
Data Sharing Statement
