# Supplementary material for: Strategies to Reduce Advanced Imaging in Antenatal Pulmonary Embolism Diagnostics
Source: JAMA Netw Open. 2025 Nov 4;8(11):e2541255. doi: 10.1001/jamanetworkopen.2025.41255 (PMC12587194; doi:10.1001/jamanetworkopen.2025.41255)
Supplement: Supplement 1. — eTable 1. Characteristics of the 2 Seminal Studies of D-Dimer–Based Algorithms for Ruling Out Pulmonary Embolism Without Advanced Imaging eTable 2. Revised and Pregnancy-Adapted Geneva Scores eMethods 1. Details of Data Collection eMethods 2. Selection of Variables for Modeling eTable 3. Median Turnaround Times of D-Dimer Testing and Ultrasonography From Order to Completion, Stratified by Diagnostic Setting eTable 4. Setting-Specific Pursuit of Advanced Imaging, Stratified by D-Dimer Values eTable 5. Pretest Probability for Acute Pulmonary Embolism and Pursuit of Advanced Imaging Among Pregnant Patients With Intermediate D-Dimer Values (≥0.5<1.0 mg/L) eTable 6. Characteristics of Pregnant Patients Who Underwent Compression Ultrasonography That Was Not Used as a Strategy to Reduce Advanced Imaging Because Advanced Imaging Was Ordered Before Compression Ultrasonography Was Ordered or Completed eResults. US Cost Estimates of Strategies to Reduce Advanced Imaging in Antenatal Pulmonary Embolism Diagnostics eTable 7. US Federal Reimbursements in Dollars for D-Dimer Testing and Compression Ultrasonography in 2022 and 2024 eTable 8. Features of 2 Strategies to Reduce Advanced Imaging in Antenatal Pulmonary Embolism Diagnostics eDiscussion. D-Dimer Use Discouraged by the 2018 American College of Obstetricians and Gynecologists Practice Bulletin [file jamanetwopen-e2541255-s001.pdf]

Supplemental Online Content

Vinson DR, Somers MJ, Zekar L, et al. Strategies to reduce advanced imaging in antenatal pulmonary embolism diagnostics. *JAMA Netw Open*. 2025;8(11):e2541255. doi:10.1001/jamanetworkopen.2025.41255

eTable 1. Characteristics of the 2 Seminal Studies of D-Dimer–Based Algorithms for Ruling Out Pulmonary Embolism Without Advanced Imaging
eTable 2. Revised and Pregnancy-Adapted Geneva Scores
eMethods 1. Details of Data Collection
eMethods 2. Selection of Variables for Modeling
eTable 3. Median Turnaround Times of D-Dimer Testing and Ultrasonography From Order to Completion, Stratified by Diagnostic Setting
eTable 4. Setting-Specific Pursuit of Advanced Imaging, Stratified by D-Dimer Values
eTable 5. Pretest Probability for Acute Pulmonary Embolism and Pursuit of Advanced Imaging Among Pregnant Patients With Intermediate D-Dimer Values (≥0.5<1.0 mg/L)
eTable 6. Characteristics of Pregnant Patients Who Underwent Compression Ultrasonography That Was Not Used as a Strategy to Reduce Advanced Imaging Because Advanced Imaging Was Ordered Before Compression Ultrasonography Was Ordered or Completed
eResults. US Cost Estimates of Strategies to Reduce Advanced Imaging in Antenatal Pulmonary Embolism Diagnostics
eTable 7. US Federal Reimbursements in Dollars for D-Dimer Testing and Compression Ultrasonography in 2022 and 2024
eTable 8. Features of 2 Strategies to Reduce Advanced Imaging in Antenatal Pulmonary Embolism Diagnostics
eDiscussion. D-Dimer Use Discouraged by the 2018 American College of Obstetricians and Gynecologists Practice Bulletin

This supplemental material has been provided by the authors to give readers additional information about their work.

© 2025 Vinson DR et al. *JAMA Network Open*.

eTable 1. Characteristics of the 2 Seminal Studies of D-Dimer–Based Algorithms for Ruling Out Pulmonary Embolism Without Advanced Imaging

	**CT-PE-Pregnancy Study**[14]	**Artemis Study**[a 15]
**Initial publication date**	Oct 23, 2018	March 20, 2019
**Patients, no**	395	498
**Mean age (SD), years**	31 (6)	31 (5)
**Gestational age, trimester (weeks), n (%)**		
First (<12)	75 (19)	74 (15)
Second (13-26)	178 (45)	193 (39)
Third (≥27)	142 (36)	231 (46)
**DVT symptoms**	59 (15)	47 (9.4)
**Previous VTE**	29 (7.3)	30 (6.0)
**Risk score**	Revised Geneva	Pregnancy-adapted YEARS
**D-dimer-based indications for advanced imaging**		
**D-dimer, mg/L** (category)		
<0.5 (low)	Indicated if revised Geneva score high (≥11)	Not indicated
>0.5<1.0 (intermediate)	Indicated	Indicated if at least 1 YEARS criterion is present
≥1.0 (high)	Indicated	Indicated
**PE ruled-out without advanced imaging, n/N (%)**	46/396 (11.6%)	195/498 (39.2%)
**90-day symptomatic VTE after initial rule-out without advanced imaging**	0 (0.0%; 95% CI, 0.0 to 1.0)	1 (0.2%; 95% CI, 0.04 to 1.2)

CI, confidence interval; DVT, deep vein thrombosis; PE, pulmonary embolism; VTE, venous thromboembolism.

a. The 3 YEARS criteria used in the Artemis algorithm are as follows: (1) PE as the most likely diagnosis, (2) clinical signs of DVT, and (3) hemoptysis. This full set of YEARS criteria could not be accurately measured retrospectively absent a prescribed protocol (which we lacked), as the likelihood variable was rarely documented. This precluded a determination of which patients with intermediate D-dimer values did and did not meet YEARS criteria for advanced imaging.

© 2025 Vinson DR et al. *JAMA Network Open*.

eTable 2. Revised and Pregnancy-Adapted Geneva Scores[36]

**Revised Geneva Score**		**Pregnancy-adapted Geneva Score**	
**Variables** (n=9)	**Points**	**Variables** (n=7)	**Points**
**Age and Medical History**		**Age and Medical History**	
Age >65 years	1	Age ≥40 years	1
Previous deep vein thrombosis or pulmonary embolism	3	Previous deep vein thrombosis or pulmonary embolism	3
Surgery under general anesthesia or fracture of the lower limbs within 1 month	2	Surgery under general anesthesia or fracture of the lower limbs within 1 month	2
Active malignant condition (solid or hematologic malignant condition, currently active or considered cured <1 year)	2		N/A
**Symptoms**		**Symptoms**	
Unilateral lower-limb pain	3	Unilateral lower-limb pain	3
Hemoptysis	2	Hemoptysis	2
**Clinical signs**		**Clinical signs**	
Heart rate		Heart rate	
75-94 beats/min	3		N/A
≥95 beats/min	5	≥110	5
Pain on lower-limb deep venous palpation and unilateral edema	4	Pain on lower-limb deep venous palpation and unilateral edema	4
**Clinical probability**	**Sum**	**Clinical probability**	**Sum**
Low	0-3	Low	0-1
Intermediate	4-10	Intermediate	2-6
High	≥11	High	≥7

© 2025 Vinson DR et al. *JAMA Network Open*.

eMethods 1. Details of Data Collection

Seven abstractors (all co-investigators) undertook manual EHR review after completing uniform training on data collection methods using a standardized computerized data collection tool, as in prior PE studies.[28,29] The principal investigator answered abstraction questions throughout the study. The following were extracted from administrative and clinical databases and pre-populated in the data collection tool: demographics, comorbidities, vital signs, laboratory, imaging results, and 90-day outcomes. Abstractors confirmed study eligibility, sites of care, and prepopulated variables. They also identified unstructured variables, e.g., the nature and duration of PE symptoms, location and extent of PE, documented use of a D-dimer-based risk instrument (YEARS, Wells, and Geneva), prophylactic, preemptive, and therapeutic anticoagulation, and consultations. We captured the most extreme vital signs in the direction of concern throughout the index diagnostic encounter but before the order time of pulmonary vascular imaging, if applicable, which would reflect the worst vital signs that may have informed advanced imaging decisions, as in prior PE research.[28,29]

eMethods 2. Selection of Variables for Modeling

In the multivariable analysis we included age (dichotomized at 40 years, as in the pregnancy-adapted Geneva score [**eTable 2, above**]), self-reported race/ethnicity, socioeconomic status, other components of the pregnancy-adapted Geneva score (history of VTE, signs or symptoms of unilateral DVT, heart rate ≥110 beats/min) and other variables we surmised might alter use of D-dimer testing or compression ultrasonography, including gestational age, syncope/presyncope, pulse oximetry <95%, site of diagnostic evaluation, and non-consent to advanced imaging. We did not include hemoptysis given its infrequency (2%) and pre-pregnancy body mass index given the high proportion of missing values (17.8%).

© 2025 Vinson DR et al. *JAMA Network Open*.

eTable 3. Median Turnaround Times of D-Dimer Testing and Ultrasonography From Order to Completion, Stratified by Diagnostic Setting

	Overall	Diagnostic Setting		
		Emergency Department	Labor and Delivery Unit	Outpatient Clinic
**VTE Diagnostic Test**				
**D-dimer testing**				
No.[a]	72,154	72,061	93	1
Min, median (IQR)	58.0 (43.0-79.0)	58.0 (43.0-79.0)	81.0 (54.5-132.0)	100 hours[b] (N/A)
**Compression ultrasonography**				
No.	229	194	26	9
Min, median (IQR)	72.5 (48.5-110.0)	71.0 (47.0-106.0)	80.5 (55.0-145.0)	10 hours[b] (1.7-22.3)

IQR, interquartile range; min, minutes; VTE, venous thromboembolism
a. Case numbers for D-dimer turnaround times in the emergency department and labor and delivery unit were taken from all patients in those departments undergoing D-dimer testing during the study period. The overall turnaround time did not include the 1 outpatient D-dimer case.
b. Turnaround times in the clinic are more intelligible if reported in hours.

eTable 4. Setting-Specific Pursuit of Advanced Imaging, Stratified by D-Dimer Values[a]

	Emergency Department N=620			Labor and Delivery Unit N=84		
		Pursuit of Advanced Imaging			Pursuit of Advanced Imaging	
	Total	Yes	No	Total	Yes	No
**Use of D-dimer**						
No	116	75 (64.7)	41 (35.3)	65	50 (76.9)	15 (23.1)
Yes	504	263 (52.2)	241 (47.8)	19	16 (84.2)	3 (15.8)
**D-dimer value, mg/L** (category)						
<0.5 (low)	154	6 (3.9)	148 (96.1)	0	0	0
≥0.5<1.0 (intermediate)	173	93 (53.8)	80 (46.2)	6	4 (66.7)	2 (33.3)
≥1.0 (high)	177	164 (92.7)	13 (7.3)	13	12 (92.3)	1 (7.7)

n (row%) throughout
a. The table does not include clinic patients (n=16) given that only 1 received a D-dimer test.

© 2025 Vinson DR et al. *JAMA Network Open*.

eTable 5. Pretest Probability for Acute Pulmonary Embolism and Pursuit of Advanced Imaging Among Pregnant Patients With Intermediate D-Dimer Values (≥0.5<1.0 mg/L)

Characteristic	Overall<br>N = 179	Pursuit of Advanced Imaging<br>Yes<br>N = 97	 No<br>N = 82
**Pretest probability**[a]	n (col %)	n (row %)	n (row %)
Low	113 (63)	56 (50)	57 (50)
Intermediate	61 (34)	37 (61)	24 (39)
High	5 (3)	4 (80)	1 (20)

a. Pretest probability for acute pulmonary embolism was calculated using the pregnancy-adapted Geneva score.[36]

eTable 6. Characteristics of Pregnant Patients Who Underwent Compression Ultrasonography That Was Not Used as a Strategy to Reduce Advanced Imaging Because Advanced Imaging Was Ordered Before Compression Ultrasonography Was Ordered or Completed

Characteristic	N=29<br>No (%)
**Unilateral signs or symptoms of DVT**	
No	25 (86)
Yes	4 (14)
**Site of evaluation**	
Emergency department	26 (90)
Labor and delivery unit	3 (10)
**Ultrasound results**	
Negative for DVT	29 (100)

DVT, deep vein thrombosis

© 2025 Vinson DR et al. *JAMA Network Open*.

eResults. US Cost Estimates of Strategies to Reduce Advanced Imaging in Antenatal Pulmonary Embolism Diagnostics

U.S. federal reimbursements in 2022 for a quantitative D-dimer test was $10.18 and for bilateral and unilateral compression ultrasonography were $195.87 and $124.24, respectively (**eTable 7**). Summed costs of 524 D-dimer tests were $5,334.32, which were associated with 230 cases of averted advanced imaging among those with low-to-intermediate D-dimer values. This equated to $23.19 per case of averted imaging, an expense significantly lower than that of advanced imaging.

U.S. federal reimbursements in 2022 for bilateral and unilateral compression ultrasonography were $195.87 and $124.24, respectively (**eTable 7**). Summed costs of 229 cases of compression ultrasonography (192 bilateral and 37 unilateral studies) were $42,204 to avert 2 cases of advanced imaging, or $21,102 per case.

Note: The American College of Radiology no longer posts the 2022 figures, as these have since been replaced with data from more recent years.[39] Recent reimbursements for compression ultrasonography are slightly lower than they were in 2022.

eTable 7. US Federal Reimbursements in Dollars for D-Dimer Testing and Compression Ultrasonography in 2022 and 2024

Diagnostic Tests	CPT Code	2022 Reimbursement	2024 Reimbursement
Quantitative D-dimer (39)	85379	10.18	10.18
Bilateral compression ultrasonography[a]	93970	195.87	184.04
Unilateral compression ultrasonography[a]	93971	124.24	116.91

a. In early 2025, 2022 data of impacts for non-70,000 series CPT codes were removed from the American College of Radiology Medicare Physician Fee Schedule webpage.[39] We have posted their 2022 spreadsheet here: https://www.kpcrest.net/acr

© 2025 Vinson DR et al. *JAMA Network Open*.

eTable 8. Features of 2 Strategies to Reduce Advanced Imaging in Antenatal Pulmonary Embolism Diagnostics

Features	D-dimer Testing	Compression Ultrasonography
Purpose in radiation-reducing strategies	To guide use of pre-test probability algorithms to safely rule-out PE without advanced imaging[17,20]	To diagnose DVT (and rule-in VTE), allowing anticoagulation without needing advanced imaging[20]
Safety of test itself	Safe for patient and fetus	Safe for patient and fetus
Different approaches guide patient selection	A. Pregnancy-adapted YEARS algorithm: for all[15]<br>B. Revised Geneva algorithm: for patients with low-to-intermediate pretest probability[14]<br>(see eTable 1 above)	A. Symptom-driven: recommended by most society guidelines[1,3,18-20]<br>B. Symptom-agnostic: can be used either first in the diagnostic pathway (pre-D-dimer) or only in those with elevated D-dimer level
Availability in ED and L&D settings in U.S. medical centers	Often available around-the-clock	May be limited after hours
Turnaround time in the APED study[a]	Approximately 60-80 minutes	Approximately 80 minutes
Cost estimates (based on federal reimbursements in the U.S. in 2022)[b]	Quantitative: $10.18	Unilateral: $124.24; bilateral: $195.87
Yield		
In the literature	A. Pregnancy-adapted YEARS algorithm: reduces need for advanced imaging by 39.2%[15]<br>B. Revised Geneva algorithm: reduces need for advanced imaging by 11.6%[14]<br>(see eTable 1 above)	Symptom-driven: DVT diagnosed in 8% of patients with DVT signs or symptoms[20]<br>Symptom-agnostic: DVT diagnosed in 1% of patients without DVT signs or symptoms[20]
In the APED study	Specific algorithms were not evaluated	Symptom-driven: DVT diagnosed in 6% of patients with DVT signs or symptoms<br>Symptom-agnostic: DVT diagnosed in 0% of patients without DVT signs or symptoms.
Number needed to test to avoid 1 advanced imaging study in the APED study	2.3	115
Estimated U.S. costs in 2022 to avert 1 advanced imaging study given use patterns observed in the APED study[b]	$23.19	$21,102.00

© 2025 Vinson DR et al. *JAMA Network Open*.

eTable 8. Features of 2 Strategies to Reduce Advanced Imaging in Antenatal Pulmonary Embolism Diagnostics

Features	D-dimer Testing	Compression Ultrasonography
Other limitations	Older D-dimer assays have lower performance	Operator dependent

APED, Antenatal Pulmonary Embolism Diagnostics; DVT, deep vein thrombosis; ED, emergency department; LDU, labor and delivery unit.
a. See **eTable 3** for turnaround times.
b. See **eResults** and **eTable 7** for U.S. cost estimates.

eDiscussion. D-Dimer Use Discouraged by the 2018 American College of Obstetricians and Gynecologists Practice Bulletin

The most recent Practice Bulletin on this topic from the American College of Obstetricians and Gynecologists (ACOG) was published in early 2018. At that time, ACOG rightly discouraged D-dimer use: "…given the minimal information gained from [D-dimer], it is not recommended as part of the evaluation of VTE in pregnancy or the postpartum period".[1] Guidelines from other professional societies published after 2019, however, have recommended use of D-dimer-based algorithms in pregnancy.[3,41]

© 2025 Vinson DR et al. *JAMA Network Open*.
