# Supplementary material for: Strategies to Reduce Advanced Imaging in Antenatal Pulmonary Embolism Diagnostics
Source: JAMA Netw Open. 2025 Nov 4;8(11):e2541255. doi: 10.1001/jamanetworkopen.2025.41255 (PMC12587194; doi:10.1001/jamanetworkopen.2025.41255)
Supplement: Supplement 2. — Data Sharing Statement [file jamanetwopen-e2541255-s002.pdf]

Data Sharing Statement

Vinson. Strategies to Reduce Advanced Imaging in Antenatal Pulmonary Embolism Diagnostics. *JAMA Netw Open*. Published November 04, 2025.
doi:10.1001/jamanetworkopen.2025.41255

Data
Data available: No
